# Fully Printed PEDOT:PSS-based Temperature Sensor with High Humidity Stability for Wireless Healthcare Monitoring

**DOI:** 10.1038/s41598-020-59432-2

**Published:** 2020-02-12

**Authors:** Yi-Fei Wang, Tomohito Sekine, Yasunori Takeda, Koji Yokosawa, Hiroyuki Matsui, Daisuke Kumaki, Takeo Shiba, Takao Nishikawa, Shizuo Tokito

**Affiliations:** Research Center for Organic Electronics, Yamagata University, 4-3-16, Jonan, Yonezawa, Yamagata, 992–8510 Japan

**Keywords:** Sensors and biosensors, Electronic devices

## Abstract

Facile fabrication and high ambient stability are strongly desired for the practical application of temperautre sensor in real-time wearable healthcare. Herein, a fully printed flexible temperature sensor based on cross-linked poly(3,4-ethylenedioxythiophene):poly(styrenesulfonate) (PEDOT:PSS) was developed. By introducing the crosslinker of (3-glycidyloxypropyl)trimethoxysilane (GOPS) and the fluorinated polymer passivation (CYTOP), significant enhancements in humidity stability and temperature sensitivity of PEDOT:PSS based film were achieved. The prepared sensor exhibited excellent stability in environmental humidity ranged from 30% RH to 80% RH, and high sensitivity of −0.77% °C^−1^ for temperature sensing between 25 °C and 50 °C. Moreover, a wireless temperature sensing platform was obtained by integrating the printed sensor to a printed flexible hybrid circuit, which performed a stable real-time healthcare monitoring.

## Introduction

Body temperature is an essential vital parameter reflecting the physiological activities. Monitoring of body temperature provides insight into human health conditions, such as cardiovascular condition, wound healing, pulmonological diagnostics, and syndromes prediction^1–4^. Therefore, the flexible temperature sensor is highly desired to realize personal healthcare devices, which enable real-time monitoring of an individual’s health state^5,6^.

Many efforts have been made to develop flexible temperature sensors, which mainly contain three types: pyroelectric detectors^7,8^, resistive temperature detectors (RTDs)^9,10^, and thermistors^11^. Among them, the thermistor which relies on the thermo-resistive effect of sensing material is widely used, due to its simple device structure, fast response, and wide sensing range^12,13^. Various thermistor materials have been developed and investigated, including composites of conductive filler with polymer, and temperature sensing conductive materials such as silver nanowire (AgNW)^14,15^, carbon nanotubes (CNTs)^16^, reduced graphene oxide (rGO)^17,18^, and poly(3,4-ethylenedioxythiophene): poly(styrenesulfonate) (PEDOT:PSS)^19,20^. However, most of these studies focused on improving the sensitivity and mechanical performance of temperature sensors, while their ambient stability, especially humidity stability, has rarely been considered. Additionally, the development of wearable temperature sensors via simple fabrication still a big challenge. Since the use of wearable devices inevitably exposed to ambient humidity, it is of great interest to develop a facile fabricated humidity-resistant temperature sensor.

In contrast to its counterparts, PEDOT:PSS has been proven as a promising candidate for the wearable temperature sensor, not only owing to its outstanding mechanical properties and turntable electrical characteristics, but also the superior in simple, patternable, and high reproducible fabrication, such as printing^21,22^. However, as a water-soluble polymer, the resistance of PEDOT:PSS also be easily affected by the environmental humidity, which would cause cross-talk with temperature^23,24^. Despite various demonstrations of PEDOT:PSS based temperature sensors, the humidity effect have rarely been clarified, and humidity stability has so far not been developed.

In this work, we developed a fully printed PEDOT:PSS-based temperature sensor which possessed excellent humidity stability. A high-performance sensing layer was achieved by crosslinking of PEDOT:PSS by (3-glycidyloxypropyl)trimethoxysilane (GOPS). Combined with fluorinated polymer passivation (CYTOP), the developed temperature sensor exhibits excellent stability in environmental humidity varied from 30% RH to 80% RH, and high temperature coefficient of resistance (TCR) of −0.77% °C^−1^ at a wide sensing range form 25 °C to 50 °C. Additionally, a wireless temperature sensing platform with the functions of on-site signal acquisition, condition, and data transmission was achieved by integrating the printed sensor to a printed flexible hybrid circuit, which performed a stable real-time body temperature monitoring.

## Results and Discussion

**Figure 1:**
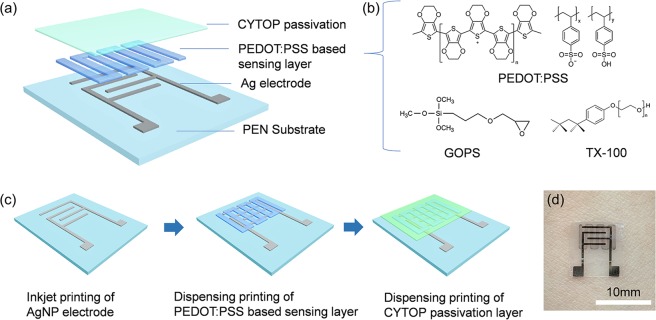
(**a**) Schematic diagram of the printed temperature sensor structure. (**b**) Chemical structure of materials for PEDOT:PSS based sensing layer. (**c**) Fabrication process of printed temperature sensor. (**d**) Photography of printed temperature sensor attached on skin.

The proposed temperature sensor was developed by a fully printing process. As a facile fabrication technique, printing not only possesses advantages in low cost and easy patterning, but also offers good uniformity and reproducibility in devices performance^25–31^. The structure of the temperature sensor was shown in Fig. 1a, which consisted of a PEN substrate, Ag electrode, temperature sensing layer, and CYTOP encapsulation layer. The temperature sensing layer was formed by PEDOT:PSS with crosslinker of GOPS, and non-ionic surfactant Triton X-100 (TX-100), as shown in Fig. 1b. The fabrication process of the fully printed temperature sensor was shown in Fig. 1c, Ag electrode (~100 nm) with digitally designed pattern was formed by inkjet printing of AgNP ink on a 50 μm PEN film, while PEDOT:PSS based thermal sensing layer (~1μm) and the CYTOP encapsulation layer (~10μm) were printed by dispenser, details are in the Experimental Section and supporting information (Figure S1). The photography of a printed temperature sensor attached to skin was shown in Fig. 1d.

As a well-known conductive polymer, PEDOT:PSS has a core-shell grain structure in which conducting PEDOT enriched cores are surrounded by the insulating PSS enriched shells^21,22^. Thus the temperature-sensitive property of the PEDOT:PSS-based temperature sensor can be explained primarily based on the enhancement of charge carrier transport and the generation of charge carriers under thermal stimulus^32–35^. As thermal energy (given temperature) increases, the limited carrier hopping and tunneling both inside the individual grain and between the neighbored nanosheets is enhanced. Therefore, the resistance of sensing film decreases with increasing the temperature, behaves a negative temperature coefficient (NTC). The sensitivity of the temperature sensor is defined by its temperature coefficient of resistance (TCR), can be calculated from Eq. (1):

1 $${TCR}=\frac{R-{R}_{0}}{{R}_{0}}\times \frac{1}{\varDelta T}\times 100 \% \,$$

where *R* and *R*_0_ are the resistance at measured temperature and room temperature (25 °C) of the sensor, ∆*T* is the change in applied temperature.

**Figure 2:**
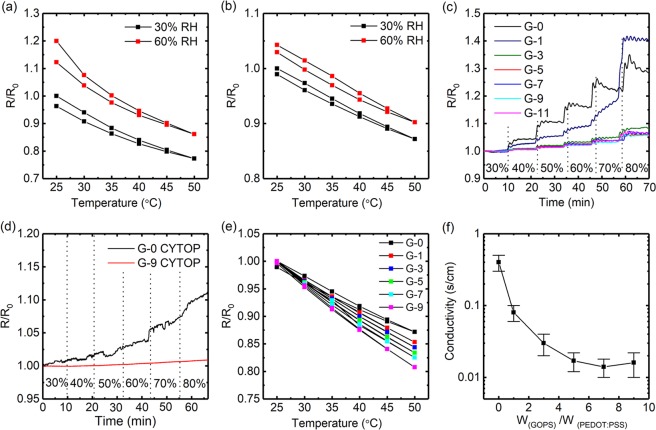
(**a**) Temperature-dependent relative resistance changes of bare PEDOT:PSS film without passivation layer, under different humidity conditions of 30% RH and 60% RH. (**b**) Temperature-dependent relative resistance changes of bare PEDOT:PSS film with CYTOP passivation layer, under different humidity conditions of 30% RH and 60% RH. (**c**) Humidity-dependent relative resistance changes of PEDOT:PSS films with different concentrations of GOPS (G-0, G-1, G-3, G-5, G-7, G-9, and G-11). (**d**) Humidity-dependent relative resistance changes of CYTOP encapsulated PEDOT:PSS films with different concentrations of GOPS (G-0, G-9). (**e**) Temperature-dependent relative resistance changes of CYTOP encapsulated PEDOT:PSS films with different concentrations of GOPS (G-0, G-1, G-3, G-5, G-7, and G-9). (**f**) Conductivity of PEDOT:PSS film with different concentrations of GOPS. Inks with GOPS to PEDOT:PSS weight ratio of 0, 1, 3, 5, 7, 9, 11 were denoted as G-0, G-1, G-3, G-5, G-7, G-9, G-11, respectively.

Due to the hydrophilic nature of PSS, the resistance of PEDOT:PSS film is susceptible to environmental humidity. The absorption of water molecules may swell PSS shells, leading to the larger distances between adjacent PEDOT enriched cores, thus increasing the resistance of film^23,24,36^. On the contrary, the humidity desorption would cause the decrease in resistance. Such effects would induce inaccuracy for temperature measurement^19,37^. At a fixed humidity condition, the sensor was equilibrium with the environment. When the sensor was heated up by thermal source (hot plate), the water molecule will be desorbed from the film (supporting information Figure S2a)^38–40^, which led to a decrease in resistance. Combined with the thermo-resistive effect induced resistance decrease, thus resulted in a higher measured TCR (supporting information Figure S2b). As shown in Fig. 2a, at a relatively low humidity state of 30% RH, temperature sensor based on bare PEDOT:PSS showed a TCR of ca. −0.91% °C^−1^, however, when humidity increased to 60% RH, resistance at 25 °C and 50 °C increased 120% and 110%, respectively, which resulting a TCR of ca. −1.14% °C^−1^. While after suppressed the humidity influence by depositing the CYTOP passivation layer, the measured TCR was decreased to ca. −0.47% °C^−1^, as shown in Fig. 2b. These results are consistent with the above discussion and revealed the importance of humidity stability for PEDOT:PSS based temperature sensors.

The fluorinated polymer CYTOP (CTX-809A) was chosen as the passivation layer, due to its low water vapor permeability and good adhesion on the substrate^41,42^. However, the resistance of the sensor still showed an apparent increase when humidity increases from 30% RH to 60% RH, while the sensitivity is relatively low for high precision detection of temperature (Fig. 2b).

To further enhance the humidity stability and temperature sensitivity of the sensor. The crosslinker of GOPS was introduced to crosslink the hydrophilic part of PSS unit. This method was used to obtain water-stable PEDOT:PSS films for organic electrochemical transistors (OECTs)^43,44^, but have yet to be studied for the temperature sensor. Since the GOPS mainly reacts with the excess PSS unit, i.e. the shell part of PEDOT:PSS grain, it will also increase the barrier at grain boundary for charge carrier hopping^45^. Therefore, the enhancements in both humidity stability and temperature sensitivity of the sensor can be expected.

Temperature sensors based on PEDOT:PSS films with different ratios of GOPS were investigated. To enable good printing performance, the surfactant TX-100 was also added. As we expected, the humidity stability of the sensors was largely improved by the adding of GOPS. Only 5% resistance increase was observed under the humidity changes for 30% RH to 80% RH when the weight ratio of GOPS to PEDOT:PSS was over 5 (Fig. 2c). After deposition of the CYTOP passivation layer, the resistance changes of temperature sensor influenced by humidity became ignorable. Thus a humidity insensitive temperature sensor was achieved, as shown in Fig. 2d.

Meanwhile, temperature sensitivity was also increased with the increase of GOPS, as shown in Fig. 2e. The optimized weight ratio of GOPS to PEDOT:PSS was 9 (ink G-9), which exhibited the highest TCR of −0.77% °C^−1^, further increasing the amount of GOPS resulted in a nonhomogeneous ink solution. The optimized thickness of CYTOP passivation deposited on the sensor is 10μm, and the effect of CYTOP thickness on TCR and humidity stability of the sensor was also investigated and showed in supporting information (Figure S3). Since the large amount of GOPS in the resulting film, the conductivity of film was dramatically decreased, as shown in Fig. 2f. By adjustment of pattern design and printing condition, the resistance of printed temperature sensor was controlled at ca. 75 KΩ, while resistance change was ca. −577.5 Ω °C^−1^, such resistance change will also benefit the circuit design and enable a high signal to noise ratio.

**Figure 3:**
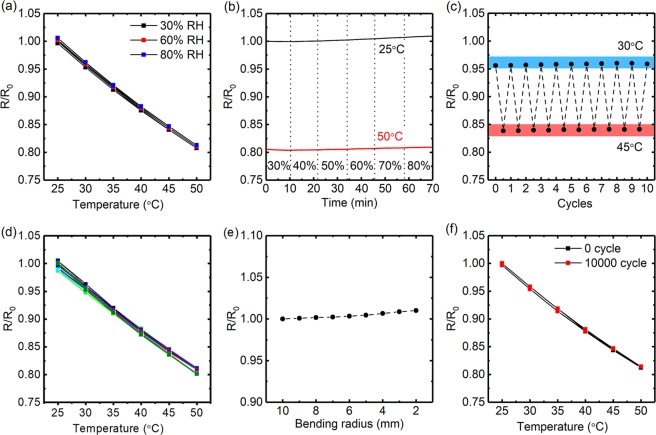
(**a**) Temperature-dependent relative resistance changes of the printed temperature sensor (ink G-9) under different humidity conditions of 30% RH, 60% RH and 80% RH, the data were obtained based on 1 cycle of heating and cooling run. (**b**) Relative-resistance changes of temperature sensor based on continuous measurement for 70 min at temperature of 25 °C and 50 °C, under humidity changes from 30% RH to 80% RH. (**c**) relative resistance changes of printed temperature sensor under cyclic heating and cooling run between 30 °C and 45 °C. (**d**) Temperature-dependent relative resistance changes of 10 printed temperature sensors. (**e**) Relative resistance changes of printed temperature sensor concerning on bending radius (from 10 to 2 mm). (**f**) Temperature-dependent relative resistance changes of printed temperature sensor before bending (0 cycle) and after cyclic bending for 10000 cycles at a bending radius of 5 mm (10000 cycles).

To evaluate the humidity stability of the developed temperature sensor, the temperature-dependent resistance changes of sensors were measured under different humidity conditions. As shown in Fig. 3a, the temperature-resistance characteristics were almost constant when the environmental humidity changes from 30% RH to 80% RH. The time-dependent resistance changes under different temperature and humidity were also monitored, as shown in Fig. 3b, the ignorable resistance change demonstrated the excellent humidity stability of our sensors. Besides humidity stability, excellent cyclic stability was also achieved. Figure 3c showed the repeatability test of temperature between 30 and 45 °C (covering skin temperature). The tiny response fluctuations indicated that the sensor performed consistently in different temperature cyclic tests. The reproducibility of the printed sensors was also characterized based on the temperature-resistance curves of 10 devices. As shown in Fig. 3d, all these sensors exhibited a similar device to device sensing performance with very slight variation in TCR from 0.75% °C^−1^ to 0.79% °C^−1^ (supporting information Figure S4), which indicated the good reproducibility of our printed sensors.

In addition, owing to the flexibility of printed electrode and PEDOT:PSS based sensing film, the sensor also exhibited excellent mechanical stability. Figure 3e showed the resistance change of the printed sensor at various bending radii (from 10 to 2 mm). Only 1% resistance increase was observed at the mentioned bending radii compared to the flat conditions. Furthermore, repetitive cyclic bending tests were also performed under 10000 bending cycles at a bending radius as small as 5 mm. No significant change in resistance was observed by gradually increasing the bending cycle (supporting information Figure S5), as well as the sensor performance (Fig. 3f).

**Figure 4:**
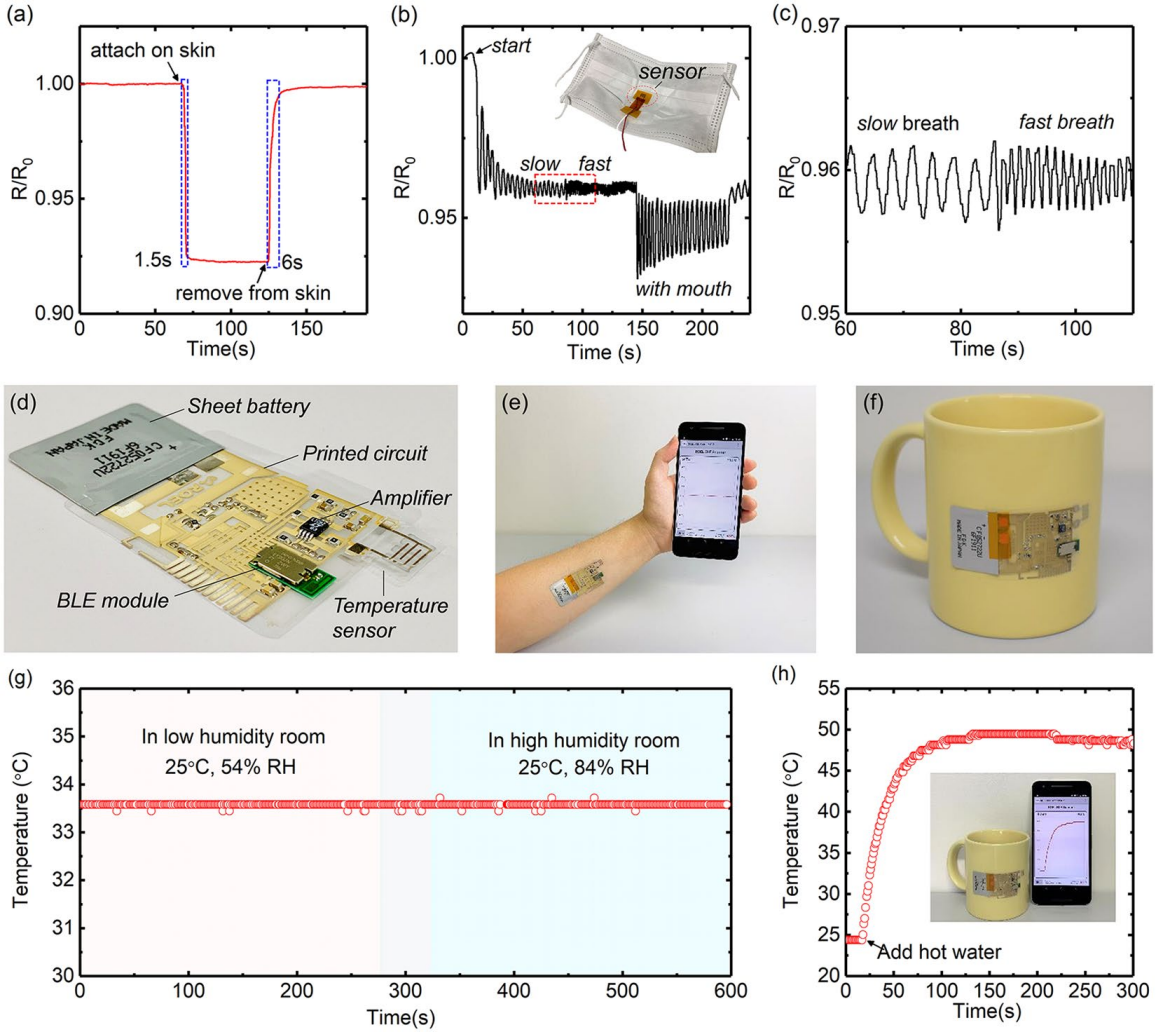
(**a**) Time-dependent resistance response of a printed temperature sensor to human skin. (**b**) Breathing rate and breathing type monitoring. (**c**) Enlarged image of date in red box region of (**b**). (**d**) Optical image of the wireless temperature sensing platform with printed temperature sensor. (**e**) Schematic diagram of wireless sensing platform mounted on an arm for real-time body temperature monitoring. (**f**) Schematic diagram of wireless sensing platform on a coffee cup for real-time object temperature monitoring. (**g**) Real-time temperature monitoring of skin by the platform under different environmental humidity. (**h**) Real-time temperature monitoring of a coffee cup after adding hot water.

The response of the printed temperature sensor to human skin temperature was shown in Fig. 4a. When the sensor was attached to the skin, its resistance decreased immediately and became stable at skin temperature value. Meanwhile, after removed from the skin, a quick increase in the resistance to the baseline was also observed. The response time (defined as the time required to move from the baseline resistance to 90% of the peak response resistance) and recovery time (defined as the time required to move from the peak response resistance to 90% of the baseline resistance) of the printed temperature sensor were estimated as 1.5 s and 6 s, respectively. Additionally, the temperature sensor also demonstrated the capability for breath monitoring. As shown in Fig. 4b, the sensor was fixed on the inner side of a mask (inset), when the volunteer wore the mask and began to breathe in such a relatively closed space, the resistance of sensor rose and fell regularly with breath in and out, respectively. Due to the excellent humidity stability of the temperature sensor, the effect of humidity during the breathing monitoring could be excluded. The breathing rate and breathing type which were firmly related to human health and activity can be obtained easily from the curve (Figs. 4b,c).

To realize real-time temperature monitoring, we developed a wireless temperature monitoring platform by integrating the printed sensor with a printed flexible hybrid circuit. As shown in Fig. 4d, the circuit was fabricated on a 50 μm thick PEN film. Multilayer flexible Ag interconnects and soft polymer insulation layer was fabricated by screen printing, while the rigid passive components, operational amplifier chip, and a Bluetooth Low Energy (BLE) module, were all assembled on the printed interconnect through surface mounted technology (SMT). The printed temperature sensor was bonded on the circuit by conductive epoxy, and a flexible battery was used as the power supply. A Bluetooth-enabled smartphone with the developed custom mobile application was used to receive, display, and store the detection results. Power consumption of the sensor is 30 µW (supporting information Figure S6), which is relatively low compared with the reported thermistor-based temperature sensors^46–48^, as shown in supporting information Table S1. Further reducing power consumption can be realized by optimizing the sensor and circuit design, which will be promoted in our future work.

Compared to the other reported flexible sensors operated by complexity measuring instruments due to the lack of flexible circuits, the presented platform can realize the on-site signal acquisition, conditioning, and wireless transmitting, which would provide superiority for the real-time on-body and on-object temperature monitoring (Figs. 4e,f). Stable body temperature recording can be achieved by the platform attached on the arm of a volunteer even the environmental humidity changed from 54% RH to 84% RH (Fig. 4g and supporting information). A sensor patch attached to the coffee cup could give clear monitoring of temperature change when adding hot water (Fig. 4h).

A comparison between our printed sensor and recently reported temperature sensors were made from the five aspects which were firmly related to practical application: the sensitivity, response time and recovery time, humidity stability, completed circuit, and fabrication method, as shown in Table 1^15,19,20,37,49,50^. It unambiguously indicated the superior comprehensive performance of our printed sensor. Moreover, the developed sensor has a wide sensing range with the maximum detection temperature of 110 °C and minimum detection temperature as low as −13 °C (supporting information Figure S7), which also offered the opportunity for the applications in robotics and the Internet of Things (IoT).

**Table 1:** Summary of some wearable temperature sensors and their performances.

Sensitive Material^a^	Sensitivity [% °C^−1^]	Response time (Recovery time)	Humidity Stability	Completed Circuit	Method	Reference
Crosslinked PEDOT:PSS	−0.77	1.5 s (6 s)	Yes	Yes	Printing	This work
AgNW	−0.33	Not given	Not given	No	Spray-coating	^15^
Gr/PEDOT:PSS	−0.06	20 s (18 s)	Not given	No	Printing	^19^
PEDOT:PSS/CNT	−0.61	18 s	Not given	No	Printing	^20^
pNIPAM /PEODT:PSS/CNT	−2.6	167 s (605 s)	No	No	Spin-coating	^37^
rGO	−0.63	7 s (25 s)	Not given	No	Wet-spinning	^49^
PEDOT:PSS	−0.25	No given	Yes	No	Printing	^50^

^a^Gr is the abbreviation for graphene, pNIPAM is the abbreviation for poly(N-isopropylacrylamide).

## Conclusion

In summary, we developed a fully printed PEDOT:PSS-based temperature sensor which possessed excellent humidity stability. The humidity effect on TCR measurement of temperature sensor was clarified. By introducing the crosslinker of (3-glycidyloxypropyl)trimethoxysilane (GOPS) and the fluorinated polymer passivation (CYTOP), the prepared sensor exhibited excellent stability in environmental humidity ranged from 30% RH to 80% RH, and high sensitivity of −0.77% °C^−1^ for temperature sensing between 25 °C and 50 °C. Moreover, a wireless temperature sensing platform was obtained by integrating the printed sensor to a printed flexible hybrid circuit, which performed a stable real-time healthcare monitoring. Such comprehensive development will undoubtedly promote the application of wearable healthcare devices.

## Methods

### Materials

PEDOT:PSS (1.3 wt% in water), Triton X-100, and (3-glycidyloxypropyl)trimethoxysilane were purchased from Sigma Aldrich, silver nanoparticle (AgNP) ink in hydrocarbon-based solution (NPS-JL) was purchased form Harima Chemicals, Fluorinated polymer CYTOP (CTX-809A) were purchased from AGC Inc. 50-μm thick poly(ethylene-phthalate) (PEN) film substrate (Q65HA) was purchased from Dupont. All materials were used as received without further purification.

### Fabrication of fully printed temperature sensor

AgNP ink was printed as electrodes using an inkjet printer (Fujifilm Dimatix, DMP2831) with 10 pl nozzles. During the inkjet printing process, the substrates and cartridge were kept at 50 and 35 °C, respectively. The substrates were then heated at 120 °C for 30 min in an air ambient to sinter the silver nanoparticles. To print the sensing layer, 100 mg PEDOT:PSS solution and 50 mg TX-100 solution (1.3 wt% in DI water) was mixed with different amount of GOPS (inks with GOPS to PEDOT:PSS weight ratio of 0, 1, 3, 5, 7, 9, 11 were denoted as G-0, G-1, G-3, G-5, G-7, G-9, G-11, respectively) by a planetary centrifugal mixer (THINKY MIXER AR-100) for 15 min (10 min mixing and 5 min degassing). The developed inks were then printed using a dispenser system (MUSASHI Engineering, Image Master 350 PC) at a pattering speed of 5 mm s^−1^ and with a discharge pressure of 2 kPa. During the dispensing process, the plates and nozzle temperatures were kept at 30 °C. Followed by an anneal at 140 °C in an air ambient for 30 min to remove the solvent and enable the cross-linking process. Finally, the encapsulation layer of CYTOP was printed by the dispenser system at 30 °C, with a pattering speed of 5 mm s^−1^ and a discharge pressure of 5 kPa, and then cured at 140 °C for 30 min. The geometry of the printed sensor was shown in supporting information Figure S1.

### Fabrication of wireless temperature sensing platform

Ag paste was screen printed on 50 μm  PEN film as interconnections of the flexible hybrid circuit, an operational amplifier (LT6004, Linear Technology), 7 chip resistors (2 mm × 1.2 mm), and 3 chip capacitors (2 mm × 1.2 mm) and a wireless BLE module (EYSGJNZWY, Taiyo Yuden Co., Ltd.) were assembled on the substrate by surface mount soldering. Further, the printed temperature sensor and a sheet-type cell battery of 25 mAh (FDK Corporation, Japan) were connected to the substrate of the circuit using conductive epoxy (CW2400, Circuit Works). Detailed experiments of design, fabrication, and optimization of the circuit will be reported elsewhere. The achieved sensor platform was attached to the skin of a volunteer using a skin-compatible adhesive.

### Measurements and characterization

The resistance change of the sensor response to the temperature was monitored by a digital multimeter (Model 34465 A, KEYSIGHT) by 4 W measurement, and recorded by a personal computer (PC) with corresponding data acquisition system. A hotplate was offered to control the measurement temperature of sensors and the temperature calibration was performed by a commercial thermocouple. A temperature and humidity chamber (SH-222, ESPEC) was used to control the temperature and humidity of the measurement environment. For the demonstration of skin temperature monitoring, the developed wireless temperature sensing platform was attached on the arm of a volunteer (Male, 30-year-old). An adhesive sheet used for tattoo paper was used here to ensure proper adhesion between the sensor patch and skin, as illustrated in supporting information Figure S8. Measurement time in high humidity condition is about 5 min. During the measurement process, the sensor patch showed a stable adhesion on the skin. An android smartphone with the developed application was used to receive and display temperature data.

Informed consent to participation in the study and publication of identified information/images in an online open-access publication was obtained from all subjects prior to their enrollment in the study. The experiments of monitoring body temperature and breath rate of a volunteer by developed sensing device were approved by the institutional review board of the Yamagata University (No. 2019–152). All the experiments that were conducted on human body in this study were performed in accordance with relevant guidelines and regulations.

## Supplementary information

Supplementary information.

Supplementary information.
