# Supplementary material for: Fully Printed PEDOT:PSS-based Temperature Sensor with High Humidity Stability for Wireless Healthcare Monitoring
Source: Sci Rep. 2020 Feb 12;10:2467. doi: 10.1038/s41598-020-59432-2 (PMC7016104; doi:10.1038/s41598-020-59432-2)
Supplement: Supplementary file 1 — Supplementary information. [file 41598_2020_59432_MOESM1_ESM.pdf]

# Supporting Information

## Fully Printed PEDOT:PSS-based Temperature Sensor with High Humidity Stability for Wireless Healthcare Monitoring

*Yi-Fei Wang, * Tomohito Sekine, Yasunori Takeda, Koji Yokosawa, Hiroyuki Matsui, Daisuke Kumaki, Takeo Shiba, Takao Nishikawa, and Shizuo Tokito **

Research Center for Organic Electronics, Yamagata University,

4-3-16, Jonan, Yonezawa, Yamagata, 992-8510, Japan

**E-mail:** wang@yz.yamagata-u.ac.jp (Y. F. Wang);

tokito@yz.yamagata-u.ac.jp (S. Tokito)

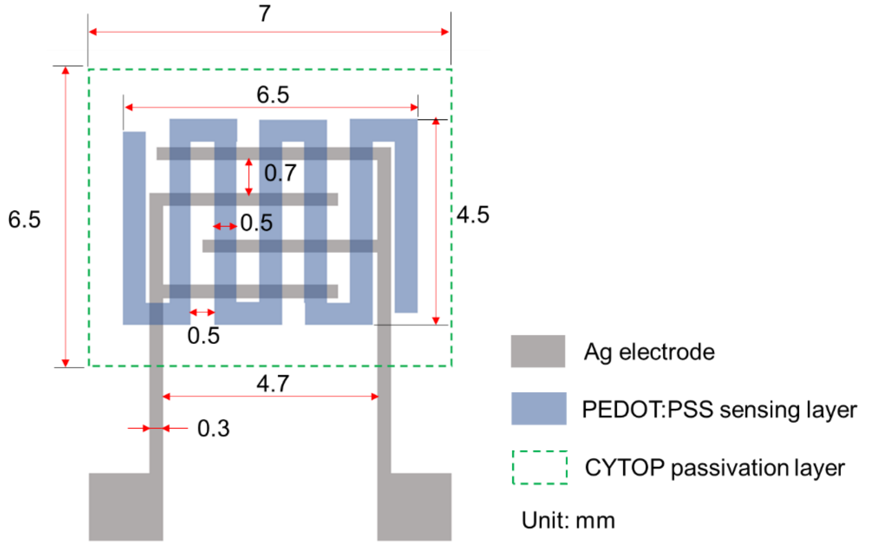


**Figure S1**. The geometry of the printed temperature sensor

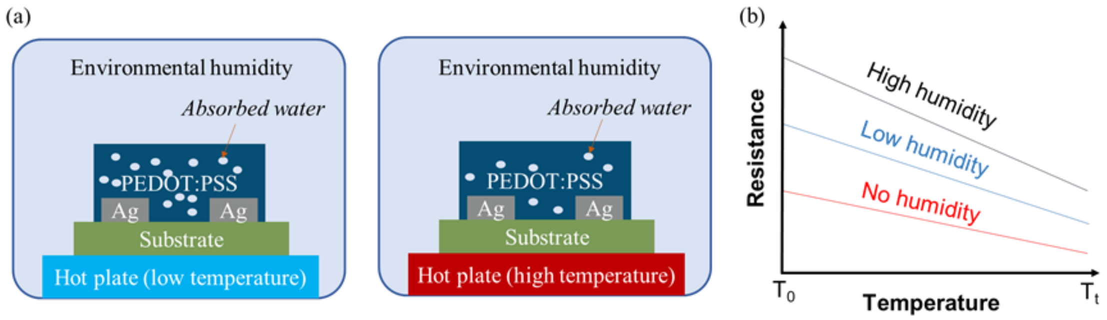


**Figure S2**. (a) illustration of humidity absorption and desorption of the temperature sensor. (b) illustration of resistance change of temperature sensor to the temperature and humidity.

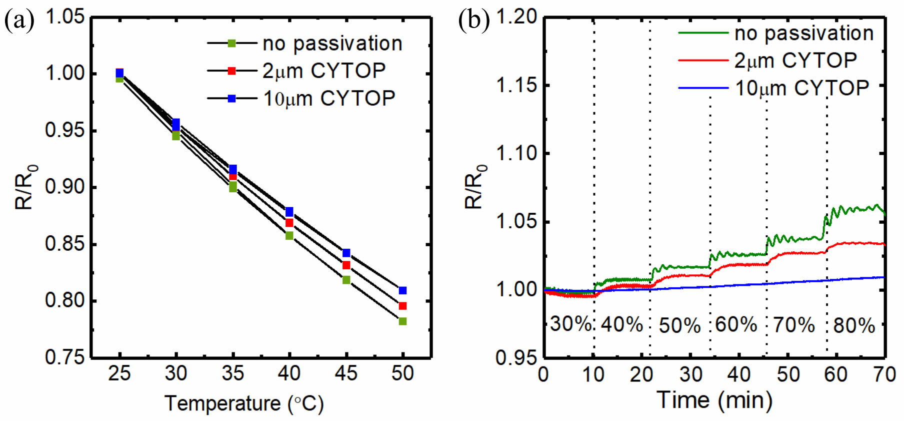


**Figure S3.** (a) Temperature-dependent relative resistance changes of temperature sensors with different thickness of passivation layers (no passivation, 2μm, and 10μm). (b) humidity-dependent relative resistance changes of temperature sensors with different thickness of passivation layers (no passivation, 2μm, and 10μm).

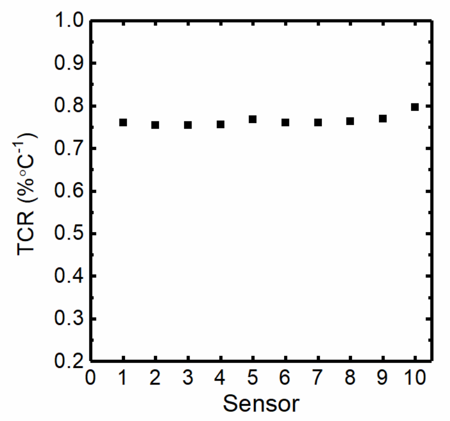


**Figure S4.** TCR of 10 printed sensors extracted from Figure 3d

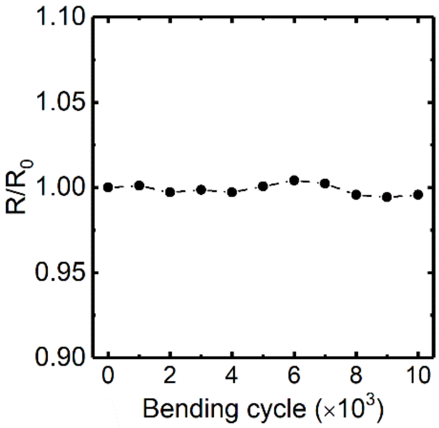


**Figure S5**. Relative resistance changes of printed temperature sensor concerning the bending cycles of up to 10000 cycles (5mm).

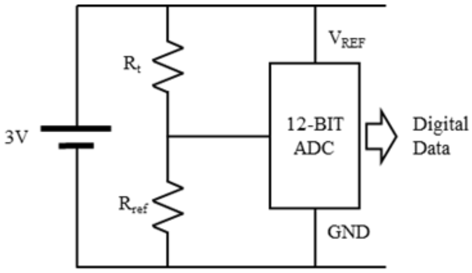


**Figure S6.** Simplified circuit diagram of the printed temperature sensor, the resistance of sensor ($R_t$) and reference resistor ($R_{ref}$) are 75KΩ,

**Table S1.** Summary of some wearable temperature sensors and their power consumption.

| Sensitive Material [a)] | Power Consumption | Device type | Reference |
|---|---|---|---|
| Crosslinked PEDOT:PSS | 30 µW | thermistor | This work |
| Mg | 41 µW | thermistor | [S1] |
| CNT yarn | 4.4 mW | thermistor | [S2] |
| Au | 1 mW | thermistor | [S3] |

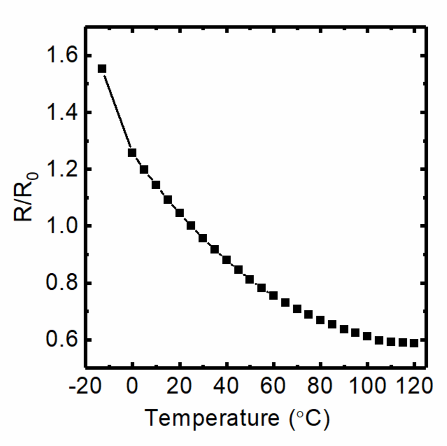


**Figure S7.** Temperature-dependent relative resistance changes of temperature sensors on extended temperature range. Sensor performance at the temperature below -13°C was not measured, due to the limitation of measurement equipment.

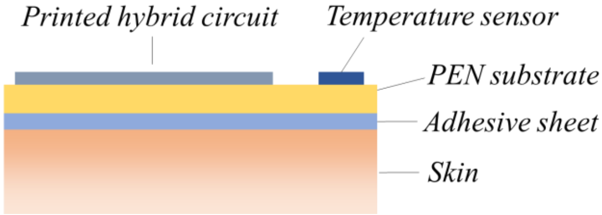


**Figure S8.** Illustration of the attachment of sensor on human skin

## References

S1. Salvatore, GA. et al. Biodegradable and Highly Deformable Temperature Sensors for the Internet of Things. *Adv. Mater.* **27**, 1702390 (2017).

S2. Dinh, T. et al. Environment-friendly carbon nanotube based flexible electronics for noninvasive and wearable healthcare. *J. Mater. Chem. C*. **4**, 10061 (2016).

S3. Webb, CR. et al. Ultrathin conformal devices for precise and continuous thermal characterization of human skin. *Nat. Mater.* **12**, 938 (2013)
